# Lessons for the clinical nephrologist: fibromuscular dysplasia in older adults

**DOI:** 10.1007/s40620-024-02039-x

**Published:** 2024-08-01

**Authors:** Henry H. L. Wu, Avanti Damle, Rajkumar Chinnadurai, Constantina Chrysochou

**Affiliations:** 1 Renal Research Laboratory, Level 9, Kolling Building, Kolling Institute of Medical Research, Royal North Shore Hospital and The University of Sydney, St. Leonards Sydney, Sydney, NSW 2065 Australia; 2 Donal O’Donoghue Renal Research Centre and Department of Renal Medicine, Northern Care Alliance NHS Foundation Trust, Salford, UK; 3 Faculty of Biology, Medicine and Health, The University of Manchester, Manchester, UK

**Keywords:** Fibromuscular dysplasia, Older adults, Geriatric nephrology, Hypertension

## The Cases

Fibromuscular dysplasia (FMD) is a non-atherosclerotic arterial disease that is a significant cause of secondary hypertension. Progression of fibromuscular dysplasia can lead to uncontrolled hypertension, renal insufficiency, strokes and rarely end-stage kidney disease. More frequently diagnosed in females and individuals between the ages of 30 and 50[1], the natural history and clinical demographics of fibromuscular dysplasia for individuals diagnosed at older age are not well understood currently.

**Fig. 1:**
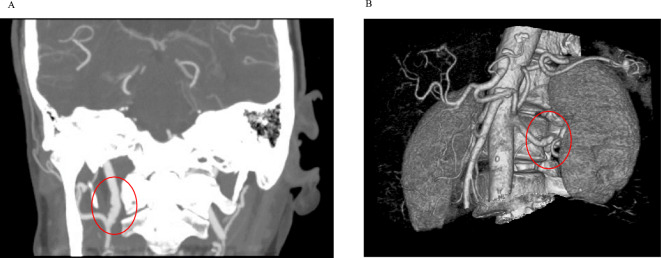
CT findings suggestive of FMD in the clinical case. **A** Internal carotid artery beading and aneurysms displayed on CT intracranial angiogram. **B** Left renal artery beading displayed in a 3-dimensional reconstruction model of the CT image. *CT* computed tomography, *FMD* fibromuscular dysplasia

A 67-year-old White female (Patient 1 in Table 1), an ex-smoker with 30-pack year history, first presented to our centre with acute onset vertigo, vomiting, increased confusion and slurring of speech in February 2020. Initial computed tomography (CT) scanning displayed a right cerebellar infarction. Further to the development of facial droop and impulsiveness, the patient had a magnetic resonance imaging (MRI) scan of her brain which showed hemorrhagic transformation with associated mass effect and distortion of the fourth ventricle alongside acute hydrocephalus, and a decompression craniectomy was then performed. In view of the subtle irregularity noted in her internal carotid arteries from the MRI, the patient proceeded to have a CT angiogram of the intracranial blood vessels which revealed internal carotid artery beading and aneurysms suggestive of fibromuscular dysplasia (Fig. 1A). A CT renal angiogram confirmed further fibromuscular dysplasia changes such as left renal artery beading (displayed in a 3-dimensional reconstruction model of the CT image in Fig. 1B). The patient was found to be hypertensive. From September 2020 onward, she has been under annual follow-up in the dedicated multidisciplinary fibromuscular dysplasia clinic at our centre. The patient was commenced on antihypertensive agents including a calcium channel blocker (Amlodipine) and a renin-angiotensin system blocker (Irbesartan). In addition, she was also commenced on a lipid lowering agent (Atorvastatin) and a lifelong antiplatelet agent (Clopidogrel). She is planned for repeat imaging of her renal vessels in 2026.

**Table 1:** Summary of cases of patients aged ≥ 65 years from the Salford FMD registry

Patient	Age/gender/ethnicity	Clinical symptoms and/or initial diagnosis on presentation	Comorbidities	Angiographic type and lesions identified on CT and/or MRI scan	Diagnosis as per FEIRI^a^	Treatment^b^
1	67 years Female White	Headache, was identified with posterior circulation stroke on presentation to hospital	Hypertension	Multifocal: left renal artery web and fusiform dilatation, irregularity of bilateral internal carotid artery, dissection of basilar artery, aneurysm of the splenic artery, stenosis at the celiac axis and ectatic common iliac artery	FMD	Medical treatment
2	69 years Female White	Pulsatile tinnitus, neck pain, burning of left side of face, was identified with transient ischaemic attack on presentation to hospital	Hypertension, hypothyroidism, hypercholesterolaemia	Unifocal: bilateral carotid stenosis, atheromatous stenosis of external iliac artery	FMD	Medical treatment
3	65 years Female White	Headache, Chest pain	Hypertension, transient ischaemic attack, SCAD	Multifocal: irregularities in renal, internal carotid and vertebral arteries	FMD + SCAD	Medical treatment and coronary angiogram
4	65 years Female White	Headache, pulsatile tinnitus, neck pain	Recurrent urinary tract infections	Multifocal: irregularities in distal main renal and bilateral carotid artery irregularity and beading. Carotid atherosclerosis	FMD	Medical treatment
5	75 years Female White	Headache, pulsatile tinnitus, was identified with stroke on presentation to hospital	Type 2 diabetes, hypertension, atrial fibrillation, stable angina	Unifocal: renal artery focal stenosis and previous left internal carotid artery para-ophthalmic aneurysm repair. Subsequently found concertina in right internal carotid artery and left middle carotid artery aneurysm. Diffuse atheroma in aorto-iliac segments	FMD	Medical treatment A left internal carotid para-ophthalmic aneurysm was treated with pipeline stent
6	66 years Female White	Chest pain, palpitations	Breast cancer, myocardial infarction	Multifocal: Irregularity, beading, dissection in the right renal artery, and left renal artery minor proximal irregularity. Irregularity of the proximal external iliac arteries bilaterally (minor) was noted	FMD + SCAD	Medical treatment
7	68 years Female White	Stroke	Hypertension, hyperthyroidism, dyslipidaemia	Multifocal: evidence of concertina and irregular vessel walls in bilateral internal carotid artery. Minor calcified disease in the celiac artery and superior mesenteric artery	FMD	Medical treatment
8	73 years Female White	Incidental diagnosis of FMD, patient was asymptomatic on presentation	Type 2 diabetes mellitus, hypertension, chronic kidney disease	Multifocal: irregular beaded stenosis of the distal right renal artery and irregularity of the left distal renal artery	FMD	Medical treatment
9	78 years Female White	Headache, pulsatile tinnitus	Type 2 diabetes mellitus, celiac disease, duodenal ulcer, glaucoma with macular degeneration	Multifocal: bilateral renal artery aneurysms. Left internal carotid artery appeared with a beaded appearance. Tortuous and beaded external iliac arteries	FMD	Medical treatment
10	80 years Female White	Incidental diagnosis of FMD, patient was asymptomatic on presentation	Aortic stenosis, Transient ischaemic attack, Hypertension	Multifocal: beading of right renal artery. Saccular aneurysm of infrarenal aorta. Irregularity of both external iliac arteries	FMD	Medical treatment

*FMD* fibromuscular dysplasia, *SCAD* spontaneous coronary artery dissection

^a^Refers to diagnostic criteria recommended by the international consensus guidelines on FMD

^b^Medical treatment refers to the prescription of antiplatelet, antihypertensive and a lipid-lowering agent, which is the standard medical treatment regimen in FMD as per current guidelines

We identified and report a further 9 cases (Patients 2–10 in Table 1) from the Salford fibromuscular dysplasia database, all of whom were aged ≥ 65 years on presentation. The mean age of this cohort was 69.5 years. All of the patients are females and of White ethnicity. Headache and pulsatile tinnitus were the chief presenting symptoms. Three of nine patients presented with a stroke, with investigations subsequently confirming a diagnosis of fibromuscular dysplasia. Along with fibromuscular dysplasia, two of nine patients were diagnosed with concurrent spontaneous coronary artery dissection (SCAD). One of these two patients had had a separate episode of spontaneous coronary artery dissection previously. Hypertension was the most frequently observed comorbidity (6 of 9 patients). Select CT and magnetic resonance angiogram images for these further cases are presented in Supplementary Fig. 1. The majority of cases identified were multifocal fibromuscular dysplasia based on the angiographic type (7 of 9 patients). Only three patients had concurrent atherosclerotic lesions detected alongside fibromuscular dysplasia. All 9 patients were managed as per the standard approach recommended by the current international consensus guidelines for the management of fibromuscular dysplasia [1]. All patients received antiplatelet treatment and renin-angiotensin system blockade on an individual risk–benefit basis, as well as being commenced on a lipid-lowering agent. All 9 patients are currently under outpatient follow-up. The median follow-up from fibromuscular dysplasia diagnosis is 21 months.

## Lessons for the clinical nephrologist

In 2005, a series of 4 cases belonging to one centre in the United States describing renal artery stenosis secondary to fibromuscular dysplasia involving unrelated female patients (aged 72, 71, 74 and 69 years, respectively) initially brought this topic to light [2]. All 4 patients presented with severely uncontrolled hypertension without kidney failure, which was acutely resolved following angioplasty and stent placement. On follow-up, all 4 patients displayed improved blood pressure control with maintenance antihypertensive treatment. Through this seminal case series, Pascual et al. [2] called into question the previously reported low prevalence of fibromuscular dysplasia in older individuals, challenging conventional assumptions that older persons with secondary hypertension would typically be attributed to atherosclerotic arterial disease only and not fibromuscular dysplasia.

It was not until the next decade that the characteristics of fibromuscular dysplasia in the older population were studied in detail in a multi-centre observational study. The two forms of fibromuscular dysplasia—focal and multifocal fibromuscular dysplasia—are described to be associated with different clinical phenotypes and appear with distinct disease entities [3]. Patients with focal fibromuscular dysplasia were previously found to be significantly younger than patients with multifocal fibromuscular dysplasia [3]. Data from the European/International fibromuscular dysplasia registry of the first 1000 patients support the mean age of diagnosis 46 ± 16 years with 12% of patients being ≥ 65 years old [4].

Bagh et al. [5] aimed to determine the differences in clinical presentation between older (i.e. age ≥ 65 years) and younger multifocal fibromuscular dysplasia patients through the evaluation of baseline demographic and clinical data in the United States registry for fibromuscular dysplasia as of December 2016. Amongst 1016 patients who were included in the observational analysis, 170 patients (16.7% of the total) were aged ≥ 65 years at the time of diagnosis. Older patients were more likely to be asymptomatic at the time of fibromuscular dysplasia diagnosis. Headache and pulsatile tinnitus, both common manifestations of fibromuscular dysplasia in the general population, were less common in older compared to younger patients. Fibromuscular dysplasia in extracranial carotid arteries was more common in patients aged ≥ 65 years at the time of diagnosis. There were no differences between older and younger patient age groups in the prevalence of renal artery involvement, number of arterial beads involved, or diagnosis of any fibromuscular dysplasia-associated aneurysms. Fibromuscular dysplasia patients aged ≥ 65 years were less likely to have had a major vascular event and undergone a therapeutic vascular procedure compared to their younger counterparts. Based on the study’s findings, Bagh et al. [5] concluded that patients with multifocal fibromuscular dysplasia aged ≥ 65 years at time of diagnosis may have had a more benign clinical phenotype and were more likely to have fewer symptoms and fibromuscular dysplasia-associated complications.

These study observations were somewhat unforeseeable, particularly the reduced prevalence of presenting complications such as arterial dissections (which includes spontaneous coronary artery dissection) in older fibromuscular dysplasia patient groups, considering such events are significantly associated with fibromuscular dysplasia and are a common mechanism resulting in fibromuscular dysplasia-associated myocardial infarction and ischaemic stroke [6]. One would expect greater risks of cardiovascular and major vascular events in older fibromuscular dysplasia patients due to hypertension being more common in older age (essential hypertension and uncontrolled hypertension are independently associated with age) [7]. Moreover, older adults are more likely to have a greater number of comorbidities and increased frailty status, both of which contribute to an increased risk of cardiovascular complications [8]. It is difficult to ascertain the true prevalence of asymptomatic aneurysms and the distribution of where such aneurysms occur, given fewer imaging studies were probably obtained for fibromuscular dysplasia cases in older adults when they were erroneously assumed to have atherosclerotic arterial disease instead. This may explain the lower frequencies of vascular events, headache, and pulsatile tinnitus that were documented compared to actual incidence.

Recently, a common genetic risk variant in the *rs9349379*—a single nucleotide polymorphism (SNP), has been found to confer an odds ratio (OR) of approximately 1.4 for fibromuscular dysplasia [9]. The same single nucleotide polymorphism variation results in an increased OR for carotid artery dissection, hypertension, migraine headache and spontaneous coronary artery dissection, all of which are associated with fibromuscular dysplasia. Interestingly, there is an inverse association with coronary atherosclerotic arterial disease and related myocardial infarction. Whilst still in the research realm, this might suggest there is less genetic susceptibility towards conventional atherosclerosis and cardiovascular events.

**Fig. 2:**
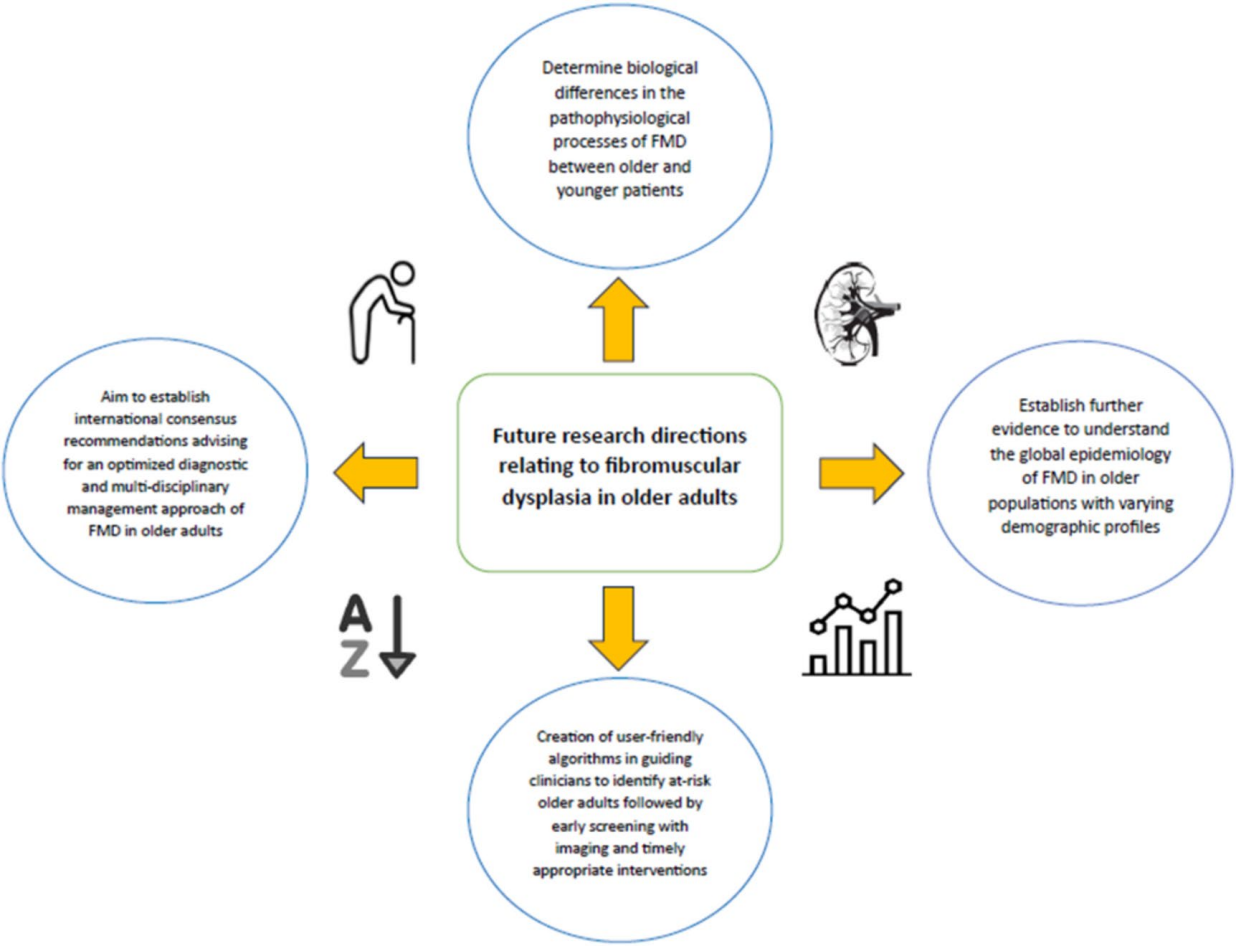
Future research directions relating to fibromuscular dysplasia in older adults. *FMD* fibromuscular dysplasia

Evaluating current evidence and uncertainties, numerous areas surrounding fibromuscular dysplasia in the older population warrant further consideration and research (Fig. 2). It remains unclear how hormonal changes and menopause may play a role in fibromuscular dysplasia. Hence, studies detailing biological changes at a basic and translational level differentiating disease processes in older compared to younger fibromuscular dysplasia patients are needed. Furthermore, a greater insight into the global epidemiology of fibromuscular dysplasia in older adults is required, based on multi-centre observational studies involving older individuals from diverse demographics and ancestry. Initiation of consensus recommendations specific to an optimised diagnostic and management approach for fibromuscular dysplasia in older populations would be ideal. It would be important to establish applicable algorithms to guide clinicians in local practice in relation to this, as timely clinical decision-making and intervention would be instrumental in lowering the risk of adverse cardiovascular and vascular complications, and improving survival outcomes.

Key messages to the clinical nephrologist:

- Early investigation of at-risk older adults presenting with uncontrolled hypertension and lower thresholds for screening with imaging tests (i.e. CT/MR angiogram) should be encouraged, to reduce false negatives and aid early diagnosis.
- Whilst current standard management of fibromuscular dysplasia involves prescription of antiplatelet agents and renin-angiotensin system blockade for blood pressure control and angioplasty in cases of resistant hypertension, perhaps the involvement of a multidisciplinary fibromuscular dysplasia care team would be more pertinent in older adults for an individualised approach in managing this uncommon condition.

## Supplementary Information

Below is the link to the electronic supplementary material.

Supplementary file1 (DOCX 305 KB)

## Data Availability

The data in this article will be shared on reasonable request to the corresponding author.
