# Supplementary material for: Lessons for the clinical nephrologist: fibromuscular dysplasia in older adults
Source: J Nephrol. 2024 Aug 1;38(1):283–8. doi: 10.1007/s40620-024-02039-x (PMC11903557; doi:10.1007/s40620-024-02039-x)
Supplement: Supplementary file 1 — Supplementary file1 (DOCX 305 KB) [file 40620_2024_2039_MOESM1_ESM.docx]

**Supplementary Fig. 1** Select CT and magnetic resonance angiogram images in older patients diagnosed with FMD

C

B

A


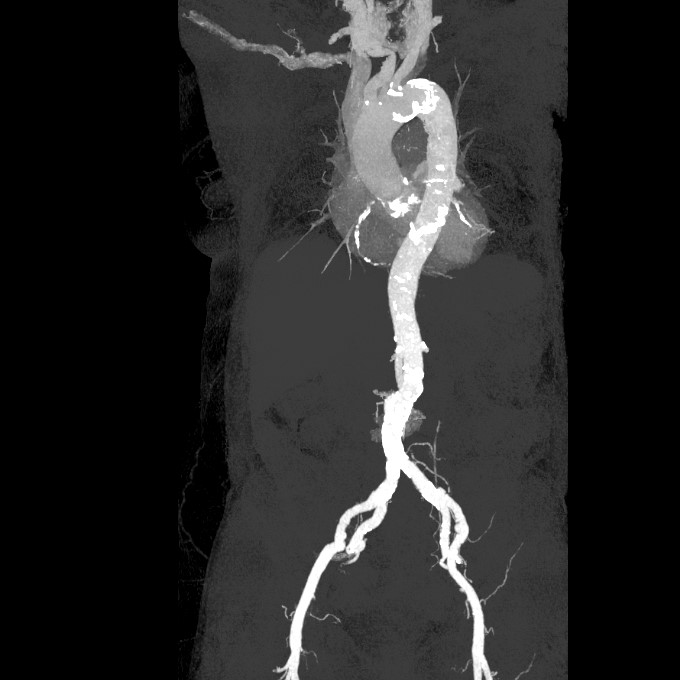
**
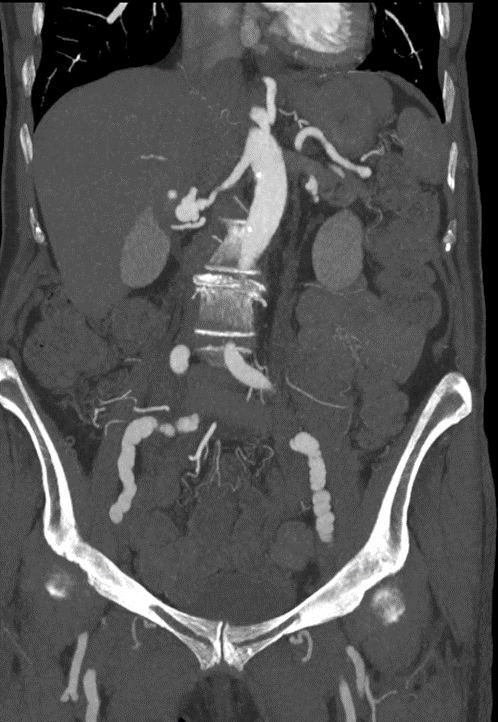

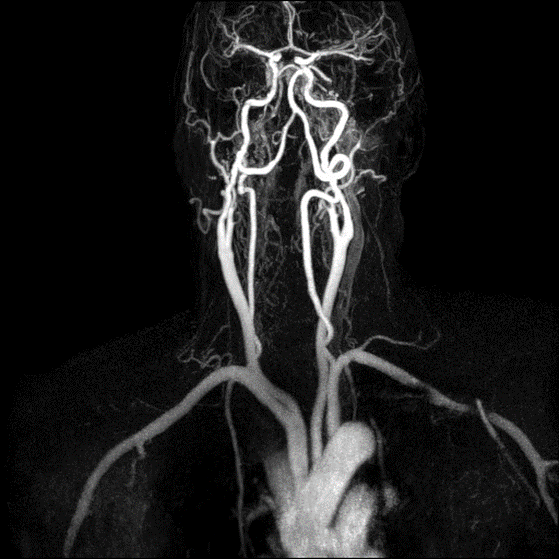
**

A: Bilateral carotid artery beading and aneurysms displayed on magnetic resonance angiogram in patient 2

B: CT angiogram displaying external iliac artery beading and bilateral renal artery aneurysms in patient 9

C: CT angiogram displaying saccular aneurysm of the infrarenal aorta in patient 10

CT: Computed tomography; FMD: Fibromuscular dysplasia
